# Antimicrobial Activity of Gepotidacin Tested against Escherichia coli and Staphylococcus saprophyticus Isolates Causing Urinary Tract Infections in Medical Centers Worldwide (2019 to 2020)

**DOI:** 10.1128/aac.01525-22

**Published:** 2023-03-06

**Authors:** S. J. Ryan Arends, Deborah Butler, Nicole Scangarella-Oman, Mariana Castanheira, Rodrigo E. Mendes

**Affiliations:** a JMI Laboratories, North Liberty, Iowa, USA

**Keywords:** ESBL, oral, resistance, urinary tract infection

## Abstract

The *in vitro* activities of gepotidacin and comparator agents against 3,560 Escherichia coli and 344 Staphylococcus saprophyticus collected from female (81.1%) and male (18.9%) patients with urinary tract infections (UTIs) in a global prospective surveillance program in 2019 to 2020 were determined. Isolates collected from 92 medical centers in 25 countries, including the United States, Europe, Latin America, and Japan, were tested for susceptibility by reference methods in a central monitoring laboratory. Gepotidacin inhibited 98.0% (3,488/3,560 isolates) of E. coli and 100% (344/344 isolates) of S. saprophyticus at gepotidacin concentrations of ≤4 μg/mL and ≤0.25 μg/mL, respectively. This activity was largely unaffected with isolates that demonstrated resistance phenotypes to other oral standard-of-care antibiotics, including amoxicillin-clavulanic acid, cephalosporins, fluoroquinolones, fosfomycin, nitrofurantoin, and trimethoprim-sulfamethoxazole. Gepotidacin also inhibited 94.3% (581/616 isolates) of E. coli isolates with an extended-spectrum β-lactamase-producing phenotype, 97.2% (1,085/1,129 isolates) of E. coli isolates resistant to ciprofloxacin, 96.1% (874/899) of E. coli isolates resistant to trimethoprim-sulfamethoxazole, and 96.3% (235/244 isolates) of multidrug-resistant E. coli isolates at gepotidacin concentrations of ≤4 μg/mL. In summary, gepotidacin demonstrated potent activity against a large collection of contemporary UTI E. coli and S. saprophyticus strains collected from patients worldwide. These data support the further clinical development of gepotidacin as a potential treatment option for patients with uncomplicated UTIs.

## INTRODUCTION

Escherichia coli is the most common pathogen causing urinary tract infections (UTIs) (1–3). Historically, these infections have been treated with oral antibiotics, including trimethoprim-sulfamethoxazole, cephalosporins, and fluoroquinolones. However, the prevalence of isolates resistant to fluoroquinolones or trimethoprim-sulfamethoxazole has increased, as has the number of isolates displaying extended-spectrum β-lactamase (ESBL)-producing phenotypes. This scenario has precluded the use of many of the aforementioned oral agents for the empirical and guided treatment of UTIs (4). This increase in ESBL-producing isolates is due in part to the rapid clonal expansion of sequence type 131 (ST131) E. coli isolates, especially in the nosocomial setting (5). ESBL-producing E. coli strains, including ST131 isolates, are often coresistant to other agents used to treat UTIs, such as fluoroquinolones, leading to the recommendation of older antibiotics for the treatment of UTIs (6). Current first-line treatment options include amoxicillin, amoxicillin-clavulanate, fosfomycin, nitrofurantoin, the amdinocillin prodrug pivmecillinam, and trimethoprim-sulfamethoxazole.

Gepotidacin is a novel, bactericidal, first-in-class triazaacenaphthylene antibacterial that inhibits DNA gyrase and topoisomerase IV by a distinct mechanism of action, which confers activity against most strains of target pathogens, such as E. coli, Staphylococcus saprophyticus, and Neisseria gonorrhoeae, including those that are resistant to current antibiotics (7, 8). Gepotidacin represents a future option for oral treatment of uncomplicated UTIs (uUTIs), defined as UTIs among premenopausal, nonpregnant women with no known urological abnormalities or comorbidities (6). Gepotidacin possesses oral bioavailability (9) and has completed to phase 3 clinical studies (ClinicalTrials.gov registration numbers NCT04020341, NCT04187144, and NCT04010539) for the treatment of uUTIs and urogenital gonorrhea (10–14). In addition, pharmacokinetic (PK)/pharmacodynamic (PD) studies and potential effects of gepotidacin on the gut microbiome have been reported (15–17).

Prior studies of gepotidacin established *in vitro* activity for the most common UTI target pathogens (18–21); however, this study prospectively monitored the *in vitro* activity of gepotidacin and comparator agents against a contemporary collection of E. coli and S. saprophyticus strains recovered from UTIs. The *in vitro* activity of gepotidacin against these isolates, as well as against subsets displaying resistance to other agents, is discussed.

## RESULTS

The gepotidacin MIC_50_ and MIC_90_ values were both 2 μg/mL against 3,560 E. coli isolates, with 98.0% of the isolates being inhibited at gepotidacin concentrations of ≤4 μg/mL (Table 1). The rates of susceptibility to amoxicillin-clavulanate (MIC_50_, 8 μg/mL; MIC_90_, 16 μg/mL), cefadroxil, ciprofloxacin (MIC_50_, 0.015 μg/mL; MIC_90_, >4 μg/mL), and trimethoprim-sulfamethoxazole (MIC_50_, ≤0.12 μg/mL; MIC_90_, >4 μg/mL) were 79.6%, 82.5%, 72.5%, and 68.2%, respectively (Table 2). Higher rates of susceptibility to fosfomycin (MIC_50_, 0.5 μg/mL; MIC_90_, 1 μg/mL; 99.0% susceptible using Clinical and Laboratory Standards Institute [CLSI] guidelines and 97.7% susceptible using European Committee on Antimicrobial Susceptibility Testing [EUCAST] guidelines), amdinocillin (MIC_50_, 0.5 μg/mL; MIC_90_, 4 μg/mL; 94.1% susceptible), nitrofurantoin (MIC_50_, 16 μg/mL; MIC_90_, 32 μg/mL; 97.3% susceptible using CLSI guidelines and 98.7% susceptible using EUCAST guidelines), and nitroxoline (99.9% susceptible) were seen for all E. coli isolates. Further stratification, based on collection setting, gender, and/or culture source, rates of susceptibility to comparator agents for countries from which at least 30 isolates were collected can be found in Tables S1 and S2 in the supplemental material for E. coli and S. saprophyticus, respectively.

**TABLE 1:** Antimicrobial activity of gepotidacin tested against E. coli and S. saprophyticus isolates collected worldwide (2019 to 2020)

Organism type^*a*^ (no. of isolates)/Phenotype	No. of isolates (cumulative %) with gepotidacin MIC (mg/L) of:											MIC_50_	MIC_90_
	≤0.03	0.06	0.12	0.25	0.5	1	2	4	8	16	32		
E. coli (3,560)	3 (0.1)	4 (0.2)	10 (0.5)	30 (1.3)	190 (6.7)	1,217 (40.8)	1,779 (90.8)	255 (98.0)	48 (99.3)	19 (99.9)	5 (100.0)	2	2
Amoxicillin-clavulanate-resistant (201)			0 (0.0)	3 (1.5)	12 (7.5)	50 (32.3)	94 (79.1)	31 (94.5)	4 (96.5)	5 (99.0)	2 (100.0)	2	4
Ciprofloxacin-resistant (899)	3 (0.3)	2 (0.6)	6 (1.2)	23 (3.8)	100 (14.9)	311 (49.5)	337 (87.0)	92 (97.2)	15 (98.9)	8 (99.8)	2 (100.0)	2	4
Fosfomycin-resistant (25)				0 (0.0)	3 (12.0)	7 (40.0)	7 (68.0)	4 (84.0)	3 (96.0)	1 (100.0)		2	8
Mecillinam-resistant (151)	1 (0.7)	0 (0.7)	0 (0.7)	3 (2.6)	8 (7.9)	39 (33.8)	78 (85.4)	17 (96.7)	3 (98.7)	2 (100.0)		2	4
Nitrofurantoin-resistant (46)			0 (0.0)	1 (2.2)	1 (4.3)	11 (28.3)	24 (80.4)	7 (95.7)	1 (97.8)	0 (97.8)	1 (100.0)	2	4
Trimethoprim-sulfamethoxazole-resistant (1,129)	2 (0.2)	0 (0.2)	5 (0.6)	12 (1.7)	87 (9.4)	420 (46.6)	468 (88.0)	91 (96.1)	31 (98.8)	9 (99.6)	4 (100.0)	2	4
Non-ESBL-producing (2,944)	1 (<0.1)	3 (0.1)	7 (0.4)	25 (1.2)	141 (6.0)	1,016 (40.5)	1,534 (92.6)	180 (98.7)	28 (99.7)	8 (>99.9)	1 (100.0)	2	2
ESBL-producing (616)	2 (0.3)	1 (0.5)	3 (1.0)	5 (1.8)	49 (9.7)	201 (42.4)	245 (82.1)	75 (94.3)	20 (97.6)	11 (99.4)	4 (100.0)	2	4
Non-MDR (3,316)	1 (<0.1)	4 (0.2)	9 (0.4)	27 (1.2)	175 (6.5)	1,133 (40.7)	1,686 (91.5)	218 (98.1)	42 (99.4)	18 (99.9)	3 (100.0)	2	2
MDR (244)	2 (0.8)	0 (0.8)	1 (1.2)	3 (2.5)	15 (8.6)	84 (43.0)	93 (81.1)	37 (96.3)	6 (98.8)	1 (99.2)	2 (100.0)	2	4
Outpatient (2,301)	0 (0.0)	3 (0.1)	6 (0.4)	15 (1.0)	122 (6.3)	763 (39.5)	1,182 (90.9)	169 (98.2)	31 (99.6)	8 (99.9)	2 (100.0)	2	2
Inpatient (1,158)	1 (0.1)	1 (0.2)	4 (0.5)	15 (1.8)	61 (7.1)	418 (43.2)	548 (90.5)	84 (97.8)	13 (98.9)	10 (99.7)	3 (100.0)	2	2
S. saprophyticus (344)	3 (0.9)	276 (81.1)	64 (99.7)	1 (100.0)								0.06	0.12

a Resistant phenotypes determined according to CLSI interpretive criteria.

**TABLE 2:** Activity of gepotidacin and comparator antimicrobial agents tested against E. coli and selected subsets

Isolate type and antimicrobial agent(no. of isolates tested)/Phenotype	MIC_50_ (μg/mL)	MIC_90_ (μg/mL)	Result with CLSI guidelines (%)^*a*^		Result with EUCAST guidelines (%)^*a*^	
			Sensitive	Resistant	Sensitive	Resistant
All E. coli isolates (3,560)						
Gepotidacin	2	2				
Ciprofloxacin	0.015	>4	72.5	25.3	72.5	25.3
Amikacin	4	8	99.5	0.3	98.2^*b*^	1.8
Amoxicillin-clavulanic acid^*c*^	8	16	79.6	5.7		
Cefadroxil	^*d*^				82.5^*e*^	17.5
Ceftriaxone	≤0.06	>8	83.7	16.1	83.7^*f*^	16.3
Fosfomycin	0.5	1	99.0^*g*^	0.7	97.7^*h*^	2.3
Mecillinam	0.5	4	94.1^*g*^	4.2	94.1^*h*^	5.9
Nitrofurantoin	16	32	97.3	1.3	98.7^*e*^	1.3
Nitroxoline	^*d*^				99.9^*e*^	0.1
Piperacillin-tazobactam^*c*^	2	8	94.7	2.8	94.7	5.3
Trimethoprim-sulfamethoxazole^*c*^	≤0.12	>4	68.2	31.8	68.2	31.3
ESBL-producing isolates (616)						
Gepotidacin	2	4				
Ciprofloxacin	>4	>4	21.5	74.6	21.5	74.6
Amikacin	4	8	97.9	1.1	91.7^*b*^	8.3
Amoxicillin-clavulanic acid^*c*^	16	32	49.1	19.7		
Cefadroxil	^*d*^				3.7^*e*^	96.3
Ceftriaxone	>8	>8	6.0	93.2	6.0^*f*^	94
Fosfomycin	0.5	2	96.6^*g*^	2.9	95.1^*h*^	4.9
Mecillinam	1	4	96.8^*g*^	2.3	96.8^*h*^	3.2
Nitrofurantoin	16	32	92.7	3.6	96.4^*e*^	3.6
Nitroxoline	^*d*^				100.0^*e*^	0.0
Piperacillin-tazobactam^*c*^	4	16	81.4	9.0	81.4	18.6
Trimethoprim-sulfamethoxazole^*c*^	>4	>4	39.8	60.2	39.8	59.5
Ciprofloxacin-resistant isolates (899)						
Gepotidacin	2	4				
Ciprofloxacin	>4	>4	0.0	100.0	0.0	100.0
Amikacin	4	8	98.4	0.8	93.8^*b*^	6.2
Amoxicillin-clavulanic acid^*c*^	8	16	60.0	9.6		
Cefadroxil	^*d*^				49.7^*e*^	50.3
Ceftriaxone	4	>8	49.6	50.2	49.6^*f*^	50.4
Fosfomycin	0.5	2	97.1^*g*^	2.2	95.2^*h*^	4.8
Mecillinam	1	4	94.7^*g*^	4.1	94.7^*h*^	5.3
Nitrofurantoin	16	32	93.5	3.7	96.3^*e*^	3.7
Nitroxoline	^*d*^				99.9^*e*^	0.1
Piperacillin-tazobactam^*c*^	4	16	85.5	7.2	85.5	14.5
Trimethoprim-sulfamethoxazole^*c*^	>4	>4	44.8	55.2	44.8	54.6
Trimethoprim-sulfamethoxazole-resistant isolates (1,129)						
Gepotidacin	2	4				
Ciprofloxacin	0.25	>4	51.7	49.3	51.7	43.9
Amikacin	4	8	98.7	0.8	95.7^*b*^	4.3
Amoxicillin-clavulanic acid^*c*^	8	16	64.2	8.7		
Cefadroxil	^*d*^				67.5^*e*^	32.5
Ceftriaxone	≤0.06	>8	68.3	31.4	68.3^*f*^	31.7
Fosfomycin	0.5	2	98.2^*g*^	1.5	96.3^*h*^	3.7
Mecillinam	1	8	90.8^*g*^	6.7	90.8^*h*^	9.2
Nitrofurantoin	16	32	95.1	2.3	97.7^*e*^	2.3
Nitroxoline	^*d*^				99.9^*e*^	0.1
Piperacillin-tazobactam^*c*^	2	16	88.8	6.2	88.8	11.2
Trimethoprim-sulfamethoxazole^*c*^	>4	>4	0.0	100.0	0.0	98.7
MDR isolates (244)						
Gepotidacin	2	4				
Ciprofloxacin	>4	>4	0.4	95.9	0.4	95.9
Amikacin	4	16	93.4	3.7	79.1^*b*^	20.9
Amoxicillin-clavulanic acid^*c*^	16	32	20.5	24.2		
Cefadroxil	^*d*^				7.8^*e*^	92.2
Ceftriaxone	>8	>8	5.3	5.3	5.3^*f*^	94.7
Fosfomycin	0.5	8	93.4^*g*^	5.7	91.8^*h*^	8.2
Mecillinam	1	8	95.1^*g*^	3.3	95.1^*h*^	4.9
Nitrofurantoin	16	64	89.8	6.6	93.4^*e*^	6.6
Nitroxoline	^*d*^				100.0^*e*^	0.0
Piperacillin-tazobactam^*c*^	8	64	53.5	22.2	53.5	46.5
Trimethoprim-sulfamethoxazole^*c*^	>4	>4	29.9	70.1	29.9	69.7

a Criteria published by CLSI (29) and EUCAST (31). Blank fields indicate no interpretive criteria, with the exception of amoxicillin-clavulanic acid (due to the 2:1 ratio, only CLSI criteria were applied).

b For UTIs.

c Amoxicillin-clavulanic acid was tested at a 2:1 ratio, piperacillin-tazobactam was tested with tazobactam at a fixed concentration of 4 μg/mL, and trimethoprim-sulfamethoxazole was tested at a 1:19 ratio.

d Susceptibility testing by disk diffusion; MICs were not determined.

e Breakpoints for uUTIs.

f Breakpoints for infections other than meningitis.

g Tested by agar dilution; UTI breakpoints.

h Tested by agar dilution; breakpoints for oral treatment of uUTIs.

Identical gepotidacin MIC_50_ and MIC_90_ values (MIC_50_, 2 μg/mL; MIC_90_, 4 μg/mL; 94.5% to 97.2% of isolates inhibited at ≤4 μg/mL) were observed among subsets of E. coli resistant to amoxicillin-clavulanate, ciprofloxacin, amdinocillin, nitrofurantoin, or trimethoprim-sulfamethoxazole. Only the fosfomycin-resistant isolates (*n* = 25) had a different gepotidacin MIC_90_ value of 8 μg/mL, with 84.0% of gepotidacin MIC values being ≤4 μg/mL (Table 1).

An ESBL-producing phenotype was observed in 616 (17.3%) of 3,560 E. coli isolates tested. Gepotidacin (MIC_50_, 2 μg/mL; MIC_90_, 4 μg/mL) activity against these isolates remained comparable to that for non-ESBL-producing E. coli isolates (MIC_50_, 2 μg/mL; MIC_90_, 2 μg/mL) (Table 1). Amoxicillin-clavulanate (MIC_50_, 16 μg/mL; MIC_90_, 32 μg/mL), cefadroxil, ciprofloxacin (MIC_50_, >4 μg/mL; MIC_90_, >4 μg/mL), and trimethoprim-sulfamethoxazole (MIC_50_, >4 μg/mL; MIC_90_, >4 μg/mL) had susceptibility rates of 49.1%, 3.7%, 21.5%, and 39.8%, respectively, against ESBL-producing E. coli isolates. However, the numbers of observed isolates susceptible to fosfomycin (96.6% using CLSI guidelines and 95.1% using EUCAST guidelines), amdinocillin (96.8%), nitrofurantoin (92.7% using CLSI guidelines and 96.4% using EUCAST guidelines), and nitroxoline (100%) remained high (Table 2).

Of all tested E. coli isolates, 899 (25.3%) and 1,129 (31.7%) were resistant to ciprofloxacin and trimethoprim-sulfamethoxazole, respectively (Table 2). The gepotidacin MIC_50_ and MIC_90_ values for these resistant populations were 2 and 4 μg/mL, respectively, similar to those for their respective susceptible population counterparts (MIC_50_, 2 μg/mL; MIC_90_, 2 μg/mL) (data not shown). Amoxicillin-clavulanate, cefadroxil, ciprofloxacin, and trimethoprim-sulfamethoxazole susceptibility rates were <70% for these resistant subsets, while fosfomycin, amdinocillin, nitrofurantoin, and nitroxoline susceptibility rates were >90% (Table 2).

A total of 244 E. coli isolates (6.9%) had a multidrug-resistant (MDR) phenotype. Gepotidacin activities for MDR (MIC_50_, 2 μg/mL; MIC_90_, 4 μg/mL) and non-MDR (MIC_50_, 2 μg/mL; MIC_90_, 2 μg/mL) isolates were similar (Table 1). Analogous to the data for ESBL-producing isolates, low susceptibility rates were seen for amoxicillin-clavulanate (MIC_50_, 16 μg/mL; MIC_90_, 32 μg/mL; 20.5% susceptible), cefadroxil (7.8% susceptible), ciprofloxacin (MIC_50_, >4 μg/mL; MIC_90_, >4 μg/mL; 0.4% susceptible), and trimethoprim-sulfamethoxazole (MIC_50_, >4 μg/mL; MIC_90_, >4 μg/mL; 29.9% susceptible) against MDR isolates. Against these isolates, susceptibility rates of >90% were observed for fosfomycin (93.4% using CLSI guidelines and 91.8% using EUCAST guidelines), amdinocillin (95.1%), nitrofurantoin (89.8% using CLSI guidelines and 93.4% using EUCAST guidelines), and nitroxoline (100.0%) (Table 2).

Isolates were stratified into outpatient and inpatient subsets based on the medical service line provided by the participating sites. Gepotidacin was active against the outpatient isolates (MIC_50_, 2 μg/mL; MIC_90_, 2 μg/mL) and inhibited 98.2% of *E. coli* isolates at ≤4 μg/mL (Table 1). Similar results were observed for gepotidacin against the inpatient isolates (MIC_50_, 2 μg/mL; MIC_90_, 2 μg/mL; 97.8% of isolates with MICs of ≤4 μg/mL). For some of the comparator agents, greater rates of susceptibility were seen for outpatient isolates, compared with the inpatient subset (amoxicillin-clavulanic acid: outpatient, 81.6%; inpatient, 75.4%; cefadroxil: outpatient, 85.3%; inpatient, 76.5%; cefazolin: outpatient, 83.0%; inpatient, 74.4%; ceftriaxone: outpatient, 86.5%; inpatient, 78.1%; ciprofloxacin: outpatient, 76.3%; inpatient, 64.4%; trimethoprim-sulfamethoxazole: outpatient, 70.5%; inpatient, 63.6%) (data not shown). The percentages of isolates susceptible to fosfomycin, amdinocillin, nitrofurantoin, and nitroxoline showed little difference (<1.0%) between outpatient and inpatient populations (data not shown).

Gepotidacin MIC_50_ and MIC_90_ values against 344 S. saprophyticus isolates were 0.06 and 0.12 μg/mL, respectively, and all observed gepotidacin MIC values were ≤0.25 μg/mL (Table 1). Most agents tested were active against this species, with susceptibility rates of >90% for trimethoprim-sulfamethoxazole (MIC_50_, ≤0.5 μg/mL; MIC_90_, ≤0.5 μg/mL; 97.1% susceptible), ciprofloxacin (MIC_50_, 0.25 μg/mL; MIC_90_, 0.5 μg/mL; 99.4% susceptible), nitrofurantoin (MIC_50_, 16 μg/mL; MIC_90_, 16 μg/mL; 100.0% susceptible), and vancomycin (MIC_50_, 1 μg/mL; MIC_90_, 2 μg/mL; 100.0% susceptible) (Table 3).

**TABLE 3:** Activity of gepotidacin and comparator antimicrobial agents tested against S. saprophyticus (*n* = 344)

Antimicrobial agent	MIC_50_ (μg/mL)	MIC_90_ (μg/mL)	Result with CLSI guidelines (%)^*a*^		Result with EUCAST guidelines (%)^*a*^	
			Sensitive	Resistant	Sensitive	Resistant
Gepotidacin	0.06	0.12				
Ciprofloxacin	0.25	0.5	99.4	0.3	99.4^*b*^	0.6
Fosfomycin	128	>256				
Nitrofurantoin	16	16	100.0	0.0	100.0^*c*^	0.0
Penicillin	0.25	0.5	3.5	96.5		
Trimethoprim-sulfamethoxazole^*d*^	≤0.5	≤0.5	97.1	2.9	97.1	1.7
Vancomycin	1	2	100.0	0.0	100.0	0.0

a Criteria published by CLSI (29) and EUCAST (31). Blank fields indicate no interpretive criteria.

b Defined as susceptible, with increased exposure.

c Breakpoints for uUTIs.

d Trimethoprim-sulfamethoxazole was tested at a 1:19 ratio.

## DISCUSSION

UTIs remain a common global health problem. Increasing resistance to oral agents, including cephalosporins, fluoroquinolones, and trimethoprim-sulfamethoxazole, have limited their use as empirical treatment (4). Current oral first-line empirical options for treating uUTIs include fosfomycin, nitrofurantoin, and amdinocillim (6). The data from this large collection of recent E. coli isolates from UTIs support these treatment options, as the proportions of all E. coli isolates that were susceptible to ciprofloxacin, cefadroxil, and trimethoprim-sulfamethoxazole were smaller (68.2% to 82.5%) than those for fosfomycin, amdinocillin, nitrofurantoin, and nitroxoline (94.1% to 99.9%). The contrast between these two drug sets was even more evident when the susceptibility rates against ESBL-producing and MDR isolates were compared. Against both ESBL-producing and MDR isolates, limited activity and low susceptibility rates (<50% susceptible) were seen for amoxicillin-clavulanate, cefadroxil, ciprofloxacin, and trimethoprim-sulfamethoxazole, while susceptibility rates of >90% were observed for fosfomycin, amdinocillin, nitrofurantoin, and nitroxoline.

The *in vitro* activities of fosfomycin, amdinocillin, nitrofurantoin, and nitroxoline against UTI E. coli strains, regardless of phenotype, have renewed interest in these old agents as oral options for treating UTIs. Nitrofurantoin is widely available and was approved by the U.S. FDA in 1954, and nitroxoline has been in clinical use in western European countries since 1962. Both agents have limitations, such as lack of PK and PD data, mainly bacteriostatic activity, and limited commercial availability (for nitroxoline) (22). Fosfomycin was introduced in Europe in the 1970s and was approved by the U.S. FDA in 1996 for single-dose treatment of uUTIs caused by E. coli or Enterococcus faecalis (23). Although fosfomycin is active *in vitro*, it has been reported that older clinical trial studies might have overestimated the clinical efficacy of fosfomycin (24); furthermore, a higher clinical cure rate with nitrofurantoin, compared with fosfomycin, has been reported (25). Finally, amdinocillin has been used for many decades for uUTIs in Nordic European countries and has shown *in vitro* stability against CTX-M-producing E. coli strains. However, clinical efficacy studies with these MDR isolates are lacking (26). Despite the potent *in vitro* activity shown by these older agents, these various limitations demonstrate the need for the clinical development of new agents (27).

Gepotidacin is currently under clinical development for the treatment of uUTIs and urogenital gonorrhea. In summary, gepotidacin demonstrated potent *in vitro* activity against a large global collection of contemporary E. coli isolates causing UTIs, inhibiting 98.0% of all E. coli isolates at MIC values of ≤4 μg/mL. Gepotidacin retained this activity against both ESBL-producing and MDR subsets, with 94.3% and 96.3%, respectively, of gepotidacin MIC values being ≤4 μg/mL. When tested against many subsets of drug-resistant E. coli phenotypes, gepotidacin maintained similar MIC_50_ and MIC_90_ values (2 and 4 μg/mL, respectively), with the single exception of fosfomycin-resistant E. coli strains, for which the gepotidacin MIC_90_ value was one doubling dilution higher at 8 μg/mL. However, this difference may be a result of the small sample size (*n* = 25). Of note, gepotidacin retained activity against isolates that were resistant to current first-line agents for uUTIs, with MIC values of ≤4 μg/mL for 84.0%, 96.7%, 95.7%, and 96.1% of isolates that were resistant to fosfomycin, amdinocillin, nitrofurantoin, and trimethoprim-sulfamethoxazole, respectively. Previous studies demonstrated that the gepotidacin concentration in urine after administration of 1,500 mg twice a day had a maximum value of 580 μg/mL between doses on day 1 and 920 μg/mL on day 4. Also, the steady-state total trough levels remained above 4 μg/mL within 12 h (15). These PK parameters indicate that the gepotidacin concentration in urine during the dosing interval remains above the MIC values for 98.0% of the E. coli isolates tested here, including resistant subsets.

Finally, gepotidacin (MIC_100_, 0.25 μg/mL) also demonstrated potent *in vitro* activity against contemporary S. saprophyticus isolates, against which older agents, such as fosfomycin, amdinocillin, and nitroxoline, lack activity. These *in vitro* data provide recent information and benchmark for gepotidacin activity prior to its clinical approval and use for treating uUTIs. As resistance to current therapy options continues to increase, these data support further clinical development of gepotidacin as a potential new agent for the treatment of uUTIs.

## MATERIALS AND METHODS

### Bacterial isolates.

A total of 3,560 E. coli isolates and 344 S. saprophyticus isolates were collected from 92 medical centers in 25 countries in 2019 to 2020 as part of the SENTRY Antimicrobial Surveillance Program. The geographic distribution of isolates included the United States (all nine U.S. Census Divisions, 45 medical centers) (2,176 isolates [55.7% overall]), Europe (17 countries, 34 medical centers) (1,252 isolates [32.1% overall]), Latin America (6 countries, 9 medical centers) (249 isolates [6.4% overall]), and Japan (4 medical centers) (227 isolates [5.8% overall]). All isolates were cultured from urine or urethral catheter samples and deemed responsible for UTI based on local criteria. Only 1 isolate per patient per infection episode was included in this study. Isolates were collected from both female (81.1%) and male (18.9%) patients. Most isolates (68.4%) were recovered from samples that had been collected from patients associated with medical service lines representing outpatient treatment, including ambulatory/outpatient, family practice, or emergency room services. Other isolates (31.6%) were cultured from patients in medical service lines suggestive of hospitalized individuals. Species identification was confirmed by standard biochemical tests and, where necessary, the matrix-assisted laser desorption ionization (MALDI) Biotyper (Bruker Daltonics, Billerica, MA, USA) according to the manufacturer’s instructions.

### Susceptibility testing.

The broth microdilution method was performed according to CLSI methods to determine susceptibility to gepotidacin and its comparator agents (28). Susceptibility to amoxicillin-clavulanate was tested at the CLSI-recommended 2:1 ratio. Susceptibility to amdinocillin and fosfomycin was determined by reference agar dilution following recommendations made by the CLSI in the M07 (28) and M100 (29) documents. The testing medium utilized was Mueller-Hinton agar, and fosfomycin testing included supplementation with glucose-6-phosphate at a final concentration of 25 μg/mL. Susceptibility to the comparators nitroxoline (30 μg) and cefadroxil (30 μg) was determined by disk diffusion following the CLSI M02 and M100 guidelines (29, 30). Disk inhibition zones and MIC values were validated by concurrently testing CLSI- and/or EUCAST-recommended quality control (QC) reference strains ATCC 25922, ATCC 27853, ATCC 29213, and ATCC 35218. All QC results were within published ranges (29). CLSI (29) and EUCAST (31) susceptibility interpretive criteria were used to determine susceptibility/resistance percentages for comparator agents. A single value was reported when susceptibility breakpoints agreed between CLSI and EUCAST guidelines (ciprofloxacin, ceftriaxone, amdinocillin, and trimethoprim-sulfamethoxazole) or when breakpoints exist for only one agency (cefadroxil and nitroxoline [EUCAST]). A single value (CLSI) was also reported for amoxicillin-clavulanate tested at a 2:1 ratio. When breakpoints differ between CLSI and EUCAST guidelines (fosfomycin and nitrofurantoin), the percentages of isolates considered susceptible with each breakpoint are labeled accordingly.

### Resistant subsets.

CLSI breakpoints were applied to define isolates with a phenotype of resistance to the following standard-of-care agents: amoxicillin-clavulanate, ciprofloxacin, fosfomycin, mecillinam, nitrofurantoin, and trimethoprim-sulfamethoxazole. The ESBL-producing phenotype was defined for E. coli as MIC values of ≥2 μg/mL for aztreonam, ceftazidime, or ceftriaxone (29). Isolates meeting these criteria can produce ESBL, have plasmid AmpC, and/or overexpress the intrinsic AmpC gene but are described here as presumptive ESBL producers. All E. coli strains were susceptible to meropenem. The MDR designation for isolates was similar to the criteria published by Magiorakos et al. (32), who define MDR as not susceptible to ≥1 agent in ≥3 antimicrobial classes. The antimicrobial classes and representative drugs used in the E. coli MDR analysis included broad-spectrum cephalosporins (ceftriaxone and ceftazidime), carbapenems (meropenem), a broad-spectrum penicillin combined with a β-lactamase inhibitor (piperacillin-tazobactam), fluoroquinolones (ciprofloxacin and levofloxacin), and aminoglycosides (gentamicin and amikacin).

## References

[1] Flores-Mireles AL, Walker JN, Caparon M, Hultgren SJ. 2015. Urinary tract infections: epidemiology, mechanisms of infection and treatment options. Nat Rev Microbiol 13:269–284. doi:10.1038/nrmicro3432. 25853778 PMC4457377

[2] Critchley IA, Cotroneo N, Pucci MJ, Jain A, Mendes RE. 2020. Resistance among urinary tract pathogens collected in Europe during 2018. J Glob Antimicrob Resist 23:439–444. doi:10.1016/j.jgar.2020.10.020. 33212286

[3] Wagenlehner FME, Bjerklund Johansen TE, Cai T, Koves B, Kranz J, Pilatz A, Tandogdu Z. 2020. Epidemiology, definition and treatment of complicated urinary tract infections. Nat Rev Urol 17:586–600. doi:10.1038/s41585-020-0362-4. 32843751

[4] Bader MS, Loeb M, Leto D, Brooks AA. 2020. Treatment of urinary tract infections in the era of antimicrobial resistance and new antimicrobial agents. Postgrad Med 132:234–250. doi:10.1080/00325481.2019.1680052. 31608743

[5] Critchley IA, Cotroneo N, Pucci MJ, Mendes R. 2019. The burden of antimicrobial resistance among urinary tract isolates of *Escherichia coli* in the United States in 2017. PLoS One 14:e0220265. doi:10.1371/journal.pone.0220265. 31821338 PMC6903708

[6] Gupta K, Hooton TM, Naber KG, Wullt B, Colgan R, Miller LG, Moran GJ, Nicolle LE, Raz R, Schaeffer AJ, Soper DE. 2011. International clinical practice guidelines for the treatment of acute uncomplicated cystitis and pyelonephritis in women: a 2010 update by the Infectious Diseases Society of America and the European Society for Microbiology and Infectious Diseases. Clin Infect Dis 52:e103–e120. doi:10.1093/cid/ciq257. 21292654

[7] Gibson EG, Bax B, Chan PF, Osheroff N. 2019. Mechanistic and structural basis for the actions of the antibacterial gepotidacin against *Staphylococcus aureus* gyrase. ACS Infect Dis 5:570–581. doi:10.1021/acsinfecdis.8b00315. 30757898 PMC6461504

[8] Bax BD, Chan PF, Eggleston DS, Fosberry A, Gentry DR, Gorrec F, Giordano I, Hann MM, Hennessy A, Hibbs M, Huang J, Jones E, Jones J, Brown KK, Lewis CJ, May EW, Saunders MR, Singh O, Spitzfaden CE, Shen C, Shillings A, Theobald AJ, Wohlkonig A, Pearson ND, Gwynn MN. 2010. Type IIA topoisomerase inhibition by a new class of antibacterial agents. Nature 466:935–940. doi:10.1038/nature09197. 20686482

[9] Barth A, Hossain M, Brimhall DB, Perry CR, Tiffany CA, Xu S, Dumont EF. 2022. Pharmacokinetics of oral formulations of gepotidacin (GSK2140944), a triazaacenaphthylene bacterial type II topoisomerase inhibitor, in healthy adult and adolescent participants. Antimicrob Agents Chemother 66:e01263-21. doi:10.1128/AAC.01263-21. 34633853 PMC8765319

[10] O'Riordan W, Tiffany C, Scangarella-Oman N, Perry C, Hossain M, Ashton T, Dumont E. 2017. Efficacy, safety, and tolerability of gepotidacin (GSK2140944) in the treatment of patients with suspected or confirmed Gram-positive acute bacterial skin and skin structure infections. Antimicrob Agents Chemother 61:e02095. doi:10.1128/AAC.02095-16. 28373199 PMC5444153

[11] Taylor SN, Morris DH, Avery AK, Workowski KA, Batteiger BE, Tiffany CA, Perry CR, Raychaudhuri A, Scangarella-Oman NE, Hossain M, Dumont EF. 2018. Gepotidacin for the treatment of uncomplicated urogenital gonorrhea: a phase 2, randomized, dose-ranging, single-oral dose evaluation. Clin Infect Dis 67:504–512. doi:10.1093/cid/ciy145. 29617982 PMC6070052

[12] ClinicalTrials.gov. 2021. A study to evaluate efficacy and safety of gepotidacin in the treatment of uncomplicated urinary tract infection (UTI): ClinicalTrials.gov identifier NCT04020341. https://clinicaltrials.gov/ct2/show/NCT04020341.

[13] ClinicalTrials.gov. 2022. Comparative study to evaluate efficacy and safety of gepotidacin to nitrofurantoin in treatment of uncomplicated urinary tract infection (UTI): ClinicalTrials.gov identifier NCT04187144. https://clinicaltrials.gov/ct2/show/NCT04187144.

[14] ClinicalTrials.gov. 2021. A study evaluating efficacy and safety of geptidacin compared with ceftriaxone plus azithromycin in the treatment of uncomplicated urogenital gonorrhea: ClinicalTrials.gov identifier NCT04010539. https://clinicaltrials.gov/ct2/show/NCT04010539.

[15] Overcash JS, Tiffany CA, Scangarella-Oman NE, Perry CR, Tao Y, Hossain M, Barth A, Dumont EF. 2020. Phase 2a pharmacokinetic, safety, and exploratory efficacy evaluation of oral gepotidacin (GSK2140944) in female participants with uncomplicated urinary tract infection (acute uncomplicated cystitis). Antimicrob Agents Chemother 64:e00199-20. doi:10.1128/AAC.00199-20. 32284384 PMC7318048

[16] Scangarella-Oman NE, Hossain M, Perry CR, Tiffany C, Powell M, Swift B, Dumont EF. 2023. Dose selection for a phase III study evaluating gepotidacin (GSK2140944) in the treatment of uncomplicated urogenital gonorrhoea. Sex Transm Infect 99:64–69. doi:10.1136/sextrans-2022-055518. 36411033 PMC9887395

[17] Nuzzo A, Van Horn S, Traini C, Perry CR, Dumont EF, Scangarella-Oman NE, Gardiner DF, Brown JR. 2021. Microbiome recovery in adult females with uncomplicated urinary tract infections in a randomised phase 2A trial of the novel antibiotic gepotidacin (GSK140944). BMC Microbiol 21:181. doi:10.1186/s12866-021-02245-8. 34130619 PMC8207760

[18] Farrell DJ, Sader HS, Rhomberg PR, Scangarella-Oman NE, Flamm RK. 2017. In vitro activity of gepotidacin (GSK2140944) against *Neisseria gonorrhoeae*. Antimicrob Agents Chemother 61:e02047-16. doi:10.1128/AAC.02047-16. 28069643 PMC5328517

[19] Flamm RK, Farrell DJ, Rhomberg PR, Scangarella-Oman NE, Sader HS. 2017. Gepotidacin (GSK2140944) in vitro activity against Gram-positive and Gram-negative bacteria. Antimicrob Agents Chemother 61:e00468-17. doi:10.1128/AAC.00468-17. 28483959 PMC5487655

[20] Jacobsson S, Golparian D, Scangarella-Oman N, Unemo M. 2018. In vitro activity of the novel triazaacenaphthylene gepotidacin (GSK2140944) against MDR *Neisseria gonorrhoeae*. J Antimicrob Chemother 73:2072–2077. doi:10.1093/jac/dky162. 29796611 PMC6927889

[21] Biedenbach DJ, Bouchillon SK, Hackel M, Miller LA, Scangarella-Oman NE, Jakielaszek C, Sahm DF. 2016. In vitro activity of gepotidacin, a novel triazaacenaphthylene bacterial topoisomerase inhibitor, against a broad spectrum of bacterial pathogens. Antimicrob Agents Chemother 60:1918–1923. doi:10.1128/AAC.02820-15. 26729499 PMC4776004

[22] Wijma RA, Huttner A, Koch BCP, Mouton JW, Muller AE. 2018. Review of the pharmacokinetic properties of nitrofurantoin and nitroxoline. J Antimicrob Chemother 73:2916–2926. doi:10.1093/jac/dky255. 30184207

[23] Derington CG, Benavides N, Delate T, Fish DN. 2020. Multiple-dose oral fosfomycin for treatment of complicated urinary tract infections in the outpatient setting. Open Forum Infect Dis 7:ofaa034. doi:10.1093/ofid/ofaa034. 32123690 PMC7036595

[24] Gardiner BJ, Stewardson AJ, Abbott IJ, Peleg AY. 2019. Nitrofurantoin and fosfomycin for resistant urinary tract infections: old drugs for emerging problems. Aust Prescr 42:14–19. doi:10.18773/austprescr.2019.002. 30765904 PMC6370609

[25] Huttner A, Kowalczyk A, Turjeman A, Babich T, Brossier C, Eliakim-Raz N, Kosiek K, Martinez de Tejada B, Roux X, Shiber S, Theuretzbacher U, von Dach E, Yahav D, Leibovici L, Godycki-Cwirko M, Mouton JW, Harbarth S. 2018. Effect of 5-day nitrofurantoin vs single-dose fosfomycin on clinical resolution of uncomplicated lower urinary tract infection in women. JAMA 319:1781–1789. doi:10.1001/jama.2018.3627. 29710295 PMC6134435

[26] Dewar S, Reed LC, Koerner RJ. 2014. Emerging clinical role of pivmecillinam in the treatment of urinary tract infection in the context of multidrug-resistant bacteria. J Antimicrob Chemother 69:303–308. doi:10.1093/jac/dkt368. 24068280

[27] Ambrose PG, Bhavnani SM, Rubino CM, Louie A, Gumbo T, Forrest A, Drusano GL. 2007. Pharmacokinetics-pharmacodynamics of antimicrobial therapy: it's not just for mice anymore. Clin Infect Dis 44:79–86. doi:10.1086/510079. 17143821

[28] Clinical and Laboratory Standards Institute. 2018. Methods for dilution antimicrobial susceptibility tests for bacteria that grow aerobically. Clinical and Laboratory Standards Institute, Wayne, PA.

[29] Clinical and Laboratory Standards Institute. 2022. Performance standards for antimicrobial susceptibility testing. M100Ed32. Clinical and Laboratory Standards Institute, Wayne, PA.

[30] Clinical and Laboratory Standards Institute. 2018. Performance standards for antimicrobial disk susceptibility tests. M02Ed13. Clinical and Laboratory Standards Institute, Wayne, PA.

[31] EUCAST. 2022. Breakpoint tables for interpretation of MICs and zone diameters, version 12.0, http://www.eucast.org/fileadmin/src/media/PDFs/EUCAST_files/Breakpoint_tables/v_12.0_Breakpoint_Tables.pdf.

[32] Magiorakos AP, Srinivasan A, Carey RB, Carmeli Y, Falagas ME, Giske CG, Harbarth S, Hindler JF, Kahlmeter G, Olsson-Liljequist B, Paterson DL, Rice LB, Stelling J, Struelens MJ, Vatopoulos A, Weber JT, Monnet DL. 2012. Multidrug-resistant, extensively drug-resistant and pandrug-resistant bacteria: an international expert proposal for interim standard definitions for acquired resistance. Clin Microbiol Infect 18:268–281. doi:10.1111/j.1469-0691.2011.03570.x. 21793988

